# The multifactorial role of HDAC9 at the maternal–fetal interface in the pathogenesis of preeclampsia

**DOI:** 10.1042/CS20242385

**Published:** 2025-12-19

**Authors:** Megan A. Opichka, Jennifer J. McIntosh, Justin L. Grobe

**Affiliations:** 1 Department of Physiology, Medical College of Wisconsin, Milwaukee, WI 53226; 2 Department of Obstetrics and Gynecology, Medical College of Wisconsin, Milwaukee, WI 53226; 3 Cardiovascular Center, Medical College of Wisconsin, Milwaukee, WI 53226; 4 Neuroscience Research Center, Medical College of Wisconsin, Milwaukee, WI 53226; 5 Department of Biomedical Engineering, Medical College of Wisconsin, Milwaukee, WI 53226; 6 Comprehensive Rodent Metabolic Phenotyping Core, Medical College of Wisconsin, Milwaukee, WI 53226

**Keywords:** histone deacetylases, preeclampsia, pregnancy

## Abstract

Preeclampsia, defined by hypertension and end organ damage after 20 weeks of gestation, remains a significant cause of maternal and fetal morbidity and mortality. This disorder has a diverse clinical presentation and is likely driven by several underlying mechanisms, many remaining poorly understood. However, there is emerging evidence that epigenetic regulators, including histone deacetylases (HDACs), may contribute to the pathophysiology of preeclampsia. Of the many HDACs, HDAC9 is particularly intriguing in the context of preeclampsia due to its decreased presence in preeclamptic placenta and prominent role in controlling trophoblast, vascular, and immune behavior, which are often dysregulated in this condition. This review focuses specifically on HDAC9, detailing its expression patterns, molecular properties, known and hypothesized targets at the maternal–fetal interface, and potential causes of dysregulation. Special emphasis is placed on its impact on trophoblast function, immune signaling, angiogenesis, and G-protein–coupled receptor pathways, which are frequently disrupted in preeclampsia. Although current evidence for altered HDAC9 expression in this disorder is confined to the placenta, its potential role in maternal physiology remains an open and important question. By integrating findings from placental biology and disorders with overlapping pathways such as cardiovascular disease and cancer research, this review aims to establish a framework for understanding how HDAC9 contributes to preeclampsia pathogenesis and to identify promising directions for future investigation and therapeutic development.

## Introduction

### General review of preeclampsia

Preeclampsia is a threatening hypertensive disorder of pregnancy with limited treatment options, and the rate of severe cases is currently rising in the United States [1,2]. It is characterized by new onset hypertension and end organ dysfunction after 20 weeks of gestation [3]. Diagnostic criteria (established by the American College of Obstetricians and Gynecologists) include proteinuria, signs of renal insufficiency, elevated liver enzymes, cerebral or visual symptoms, low platelet counts, and pulmonary edema [3,4]. Other common impediments include endothelial dysfunction, an exacerbated immune response, intrauterine growth restriction, and hemolysis [5,6]. Though many of these overt symptoms resolve within 12 weeks following delivery [7], maternal endothelial damage persists [8], and a history of preeclampsia poses an increased risk for future cardiac event and stroke [9–11]. In addition to the substantial maternal threat, children from pregnancies affected by preeclampsia are more susceptible to cognitive delays, cardiovascular disease, and metabolic syndrome later in life [12,13].

The etiology of preeclampsia is not well understood despite being a major cause of global maternal and fetal mortality [14]. However, it is widely accepted that the placenta is involved in the pathophysiology regardless of the initiating factor, which is likely heterogeneous [15] as profiling the maternal plasma proteome throughout gestation [16] and placenta transcriptomic signatures upon delivery [17] support the idea of many molecular subtypes. Preeclampsia is sometimes categorized based on time of onset (early < 34 weeks, late >34 weeks), with late-onset representing more than 80% of cases [18,19], but the mechanisms of each have yet to be clearly differentiated and may overlap. In both forms, it is understood that early stages encompassing placental stress [20,21] and circulating maternal changes [22,23] precede the onset of clinical features. Related to this notion, Redman, Staff, and Roberts have proposed a unifying hypothesis known as the ‘syncytiotrophoblast stress theory of preeclampsia’. This theory acknowledges that syncytiotrophoblasts, the cell comprising the outer villous layer of placenta, are exposed to various insults throughout pregnancy and accumulate generalized cellular stress. Consequently, this cell population releases bioactive factors into the maternal circulation, termed ‘syncytial stress signals’. Thus, regardless of the initiating factor, symptoms of preeclampsia occur when these syncytial stress signals surpass the capacity of the maternal systems to buffer them.

Early-onset preeclampsia is distinct in that it is more frequently accompanied by intrauterine growth restriction [19], poses a greater risk for future cardiovascular events [24], and displays a more pronounced angiogenic imbalance [25]. Early manifestations are likely caused by defective placentation [20,26], which often presents as shallow endovascular invasion of extravillous trophoblasts into the maternal spiral arteries, leading to inadequate spiral artery remodeling and placental malperfusion [26–28]. While this malperfusion was traditionally attributed to placental hypoxia due to insufficient oxygen delivery, it is now understood to also involve high-pressure, turbulent blood flow that can damage the chorionic villi [20,29]. Beyond this well-studied aspect, placental development may be impaired due to early factors such as insufficient expansion of extravillous cytotrophoblasts or premature resolution of the spiral artery plug, potentially imposing excess oxidative stress [26]. Conversely, late-onset preeclampsia may be driven by factors such as compression of the chorionic villi from placenta overgrowth or cell senescence [26]. Other contributors, not entirely restricted to either subtype but more frequently discussed and prominent in early-onset forms, include inadequate trophoblast differentiation, release of necrotic syncytiotrophoblast microparticles into maternal circulation, and placental secreted anti-angiogenic factors [19,30,31]. Preeclampsia is also attributed to increased G-protein-coupled receptor (GPCR) signaling in response to pressure-regulating hormones that can alter placental development, induce oxidative stress markers, and facilitate increased vascular resistance [32–37]. In each of the aforementioned scenarios, the subsequent cascade of aberrant physiological events present in women with this disorder is partially a consequence of placental stress, and histone deacetylases (HDACs) are related to a number of these processes [34,38–40].

### HDACs in preeclampsia: exploring the specific role of HDAC9

Epigenetic changes alter gene expression without directly modifying the DNA sequence and have a plausible role in the development of preeclampsia [41–44]. Of the many epigenetic regulators, HDACs are a type of histone modifying enzyme responsible for deacetylation of lysine residues within the histone tail. Removal of the neutralizing acetyl group [45] allows a tighter configuration between positively charged lysine and negatively charged DNA, thereby preventing RNA polymerase-mediated transcription [42,46]. In addition to this classical role, HDACs can modulate acetylation changes on non-histone proteins, affecting their structure, localization, activity, and susceptibility to phosphorylation or ubiquitination [47,48]. HDACs are generally associated with transcriptional downregulation [49,50] but can also serve as transcriptional activators [34,49,51], with one instance of this noncanonical role specifically delineated in trophoblasts within the context of preeclampsia [34].

HDACs are traditionally categorized into four classes based on their homology to yeast counterparts: Class I (HDAC1, HDAC2, HDAC3, HDAC8), IIa (HDAC4, HDAC5, HDAC7, HDAC9), IIb (HDAC6, HDAC10), III (sirtuins, a distinct group with no sequence similarity and NAD+as a cofactor rather than zinc [52]), and IV (HDAC11) [53]. This review focuses specifically on the IIa subgroup, with a particular emphasis on HDAC9, due to its emerging relevance in the placenta during preeclampsia [34,40]. Reanalysis of a large microarray dataset (GSE75010) conducted by our lab indicated HDAC9 was the only IIa family member decreased in placenta from preeclamptic pregnancies [34], with reduced mRNA and protein also reported by Xie et al. [40]. Deeper mechanistic studies by both groups revealed placenta-specific targets relevant to preeclampsia [34,40]. Xie et al. showed HDAC9 represses *TIMP3*, affecting trophoblast invasion [40]. Perschbacher et al. demonstrated HDAC9-dependent transcription of *RGS2*, a negative regulator of G-protein-coupled receptor signaling, with placental-specific loss of *Rgs2* recapitulating hallmarks of the disorder in vivo [34]. While the class I HDAC family members, HDAC3 and HDAC8, were also diminished in GSE75010, these enzymes are ubiquitously expressed and function exclusively in the nucleus [53]. In contrast, IIa enzymes, including HDAC9, are more tissue-specific and capable of shuttling between the nucleus and cytoplasm, allowing them to regulate both nuclear and cytoplasmic targets [53,54]. This dual localization and tissue specificity make class IIa HDACs particularly interesting in placental biology and preeclampsia. More recently, decreased levels of HDAC4 [55] and HDAC5 [56] have been reported in preeclamptic placentas, suggesting a broader disruption of IIa HDACs may contribute to the pathogenesis of this disorder. These findings warrant further investigation into the role of class IIa HDACs in placental function and the molecular mechanisms underlying preeclampsia.

HDAC9 exhibits an array of functions, detailed below, that influence the cell cycle, invasion, apoptosis, angiogenesis, and inflammation [54]. Accordingly, in addition to the identified placental targets [34,40], HDAC9 has been widely studied in the context of cancer and cardiovascular disease but has not been exclusively limited to these disease states [57–61]. Preeclampsia shares many features of cardiovascular disease, including endothelial dysfunction [62,63], inflammation [64,65], RAAS dysregulation [33,66–69], and angiogenic imbalance [30,70,71], all of which contribute to hypertension and impaired vascular remodeling [69,72–76]. In contrast, cancer involves some of the same molecular pathways as preeclampsia, with opposite biological outcomes often being deemed favorable. For instance, cancer progression is supported by excess angiogenesis, invasion, and immune evasion [77], whereas preeclampsia is characterized by insufficient trophoblast invasion [26–28], reduced angiogenesis [76], and immune over-activation [78]. This divergence underscores how overlapping signaling networks can drive distinct pathologies depending on environmental and cellular context, but also suggests that findings from other disease states may offer valuable insights into the molecular underpinnings of preeclampsia. Therefore, the purpose of this review is to synthesize the available literature applicable to placental HDAC9 in preeclampsia, with the aim of informing future research and therapeutic strategies. Although HDAC9 is localized in maternal tissues as well [54,79–81], current evidence of its altered expression is limited to the placenta [34,40]; thus, this work focuses primarily on the potential consequences of attenuated placental HDAC9 and interacting cells at the maternal–fetal interface. Further studies are needed to determine whether changes in maternal HDAC9 occur and influence the vascular, immune, or hormonal adaptations to pregnancy. After summarizing the general expression patterns and molecular properties of HDAC9, the following sections will outline its role in modulating trophoblast function, immunity, angiogenesis, and G-protein-coupled receptor signaling, along with potential contributors to downregulated placental HDAC9 in preeclampsia and the therapeutic implications of its activation.

## Overview of HDAC9: expression patterns, structure, and regulation

### General expression patterns

HDAC9, a class IIa family member [82], is most highly expressed in the brain but also present in a broad range of cell types including skeletal muscle, cardiac, adipose, kidney, liver, colon, thymus, lung, vascular, germ, placental, and immune cells [54,79–81]. Transcriptomic profiling of first trimester placenta suggests the highest expression in Hofbauer cells, endothelial cells, and fibroblasts, with some expression in extravillous trophoblast and cytotrophoblasts [81,83]. At term, HDAC9 immunostaining was most apparent in the syncytiotrophoblast, reflecting a potential gestational-age-dependent shift in expression patterns [40]—though some differences may be attributed to variations in RNA and protein levels, along with methodological factors, including the sensitivity and spatial resolution of both techniques. Within the immune compartment, HDAC9 expression is most prominent in dendritic cells, B cells, and monocytes, with lower levels in natural killer and T cells [81,84,85]. Accordingly, HDAC9 has been widely studied in the context of metabolic disease [54,57,86], cardiovascular disease (with an emphasis on atherosclerosis and cardiac hypertrophy) [54,57,86,87], immunity [54,57,86,87], and cancer [54,57,86], with a more recent appreciation of its relevance in placental biology [34,40].

### Structure of HDAC9 and regulation of cellular localization

**Figure 1:**
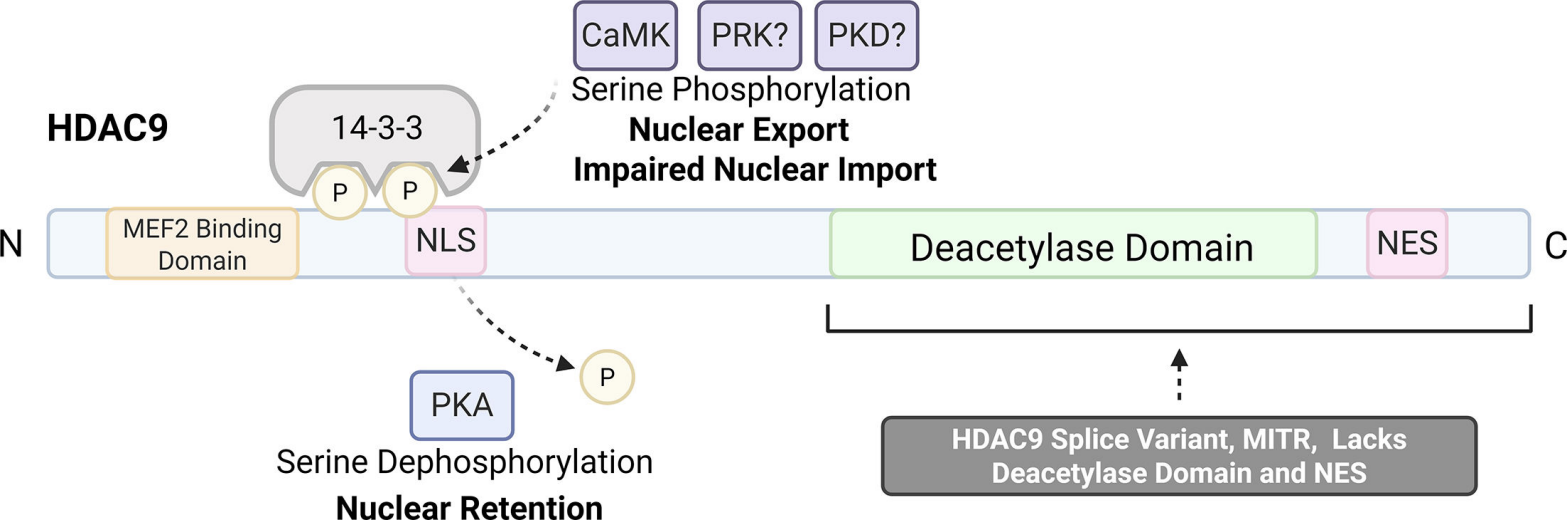
General properties of HDAC9. Structurally, HDAC9 consists of an N-terminal domain that contains binding sites for MEF2 and other proteins, along with an NLS [82,86,92]. Serine residues within this region are subject to phosphorylation events, which allows an association with 14-3-3 [82]. The interaction between class IIa HDACs and 14-3-3 maintains a cytosolic presence where HDACs are prone to degradation by i) masking the NLS or ii) causing a conformational shift thereby exposing the NES [82,86,89]. MITR is a splice variant of HDAC9 lacking the C-terminal domain [93]. CaMK is known to phosphorylate MITR, thereby promoting retention in the cytoplasm [88,94], and conserved sites with other class IIa HDACs suggest that PRK and PKD can mediate a similar effect [88,91,95]. Conversely, PKA prevents nuclear export via a downstream serine dephosphorylation event [82,88,96–98]. The C-terminal domain contains a NES and the deacetylase domain, which can interact with complexes and class I HDACs to enhance its catalytic activity [82,99]. Overall, HDAC9 has a diverse set of interactions that render it capable of controlling a broad range of cellular functions. CaMK, calcium/calmodulin-dependent protein kinase; HDAC9, histone deacetylase 9; MEF2, myocyte enhancer factor 2; NES, nuclear export signal; NLS, nuclear localization signal; MITR, MEF2-interacting transcriptional repressor; PRK, protein kinase C-related kinase; PKA, protein kinase A; PKD, protein kinase D.

Structurally, class IIa HDACs, including HDAC9, are comprised of a catalytic C-terminal domain with a nuclear export sequence (NES), a N-terminal adaptor region harboring a nuclear localization sequence (NLS), and binding sites for interacting proteins [86,88]. The deacetylase activity of class IIa is lower than class I [46,82]. Instead, proteins of the IIa subtype exert their effects by interacting with transcription factors and corepressors as well as class I HDACs, detailed in Martin et al. [89]. Full-length HDAC9 and its truncated splice variant, MEF2-interacting transcriptional repressor (MITR), which lacks the C-terminal deacetylase domain and NES, are both expressed in the placenta [54,79] and undergo post-translational modifications that alter their cellular distribution and transcriptional activity [54,88,90,91]. For instance, phosphorylation of serine residues in the N-terminal region establishes docking sites for 14-3-3 [54,82,89]. 14-3-3 enables HDAC shuttling to the cytoplasm by concealing the NLS, which would normally be identified by importinα, or by causing a conformational shift to reveal the NES [54,82,86,89]. HDAC9/MITR contains sites for regulation of shuttling by CaMK, PKD, PRK1, and other kinases (Figure 1), further reviewed by Mathias et al. [88]. Of these, CaMK and PKD influence the cellular distribution of class IIa HDACs in response to angiotensin II and endothelin-1 [100]. Notably, preeclamptic individuals exhibit heightened plasma and placental endothelin-1 levels [101,102] as well as exacerbated angiotensin II signaling. This augmented signaling is characterized by increased vascular responsiveness [33], increased angiotensin II peptide and AT1 receptors in chorionic villi [67,103], and the presence of agonistic autoantibodies targeting the AT1 receptor [104–106], which have known pathological consequences [74,75]. Thus, it is plausible that aberrant kinase activity downstream of AT1 and endothelin-1 receptor activation contributes to reduced nuclear HDAC9 in preeclampsia, potentially altering gene expression programs critical for placental function. This hypothesis, along with other suggested regulators of HDAC9 in preeclampsia, will be discussed in the subsection titled *GPCR-Associated Kinases and Oxidative Stress as Potential Drivers of HDAC9 Dysregulation in Preeclampsia*.

## Regulation of trophoblast function

### Overview of trophoblast subtypes

Trophoblasts comprise the major cell type of the placenta, and implantation and placentation are coordinated by the actions of several trophoblast subtypes [107]. These epithelial cells arise from the outer layer of the blastocyst [107,108] and differentiate into syncytiotrophoblast and extravillous lineages from cytotrophoblasts [107]. Syncytiotrophoblasts form the outer multinucleated layer of chorionic villi and are essential for villous exchange [107], hormone production [107], evading infection [109,110], and creating a protective barrier between the maternal immune system and her allogenic fetus [26]. Extravillous trophoblasts are more invasive in nature and recognized for their role in spiral artery remodeling. Endovascular extravillous trophoblasts initially form a plug within the arteries, maintaining a low-oxygen environment that supports early placental development [29,111], while interstitial extravillous trophoblasts migrate through the decidua, interacting with maternal immune cells to help reconstruct the vessel walls from the outside [111,112]. Some extravillous trophoblasts also give rise to multinucleated giant cells and contribute to glandular remodeling, enhancing nutrient availability [111,113]. Obstetric complications, including preeclampsia, may be ascribed to early defects in placentation or the accretion of placental stress caused by the inherent demands of pregnancy and a compilation of internal and external determinants [26].

### HDAC9 targets and their role in trophoblast development and function

**Figure 2:**
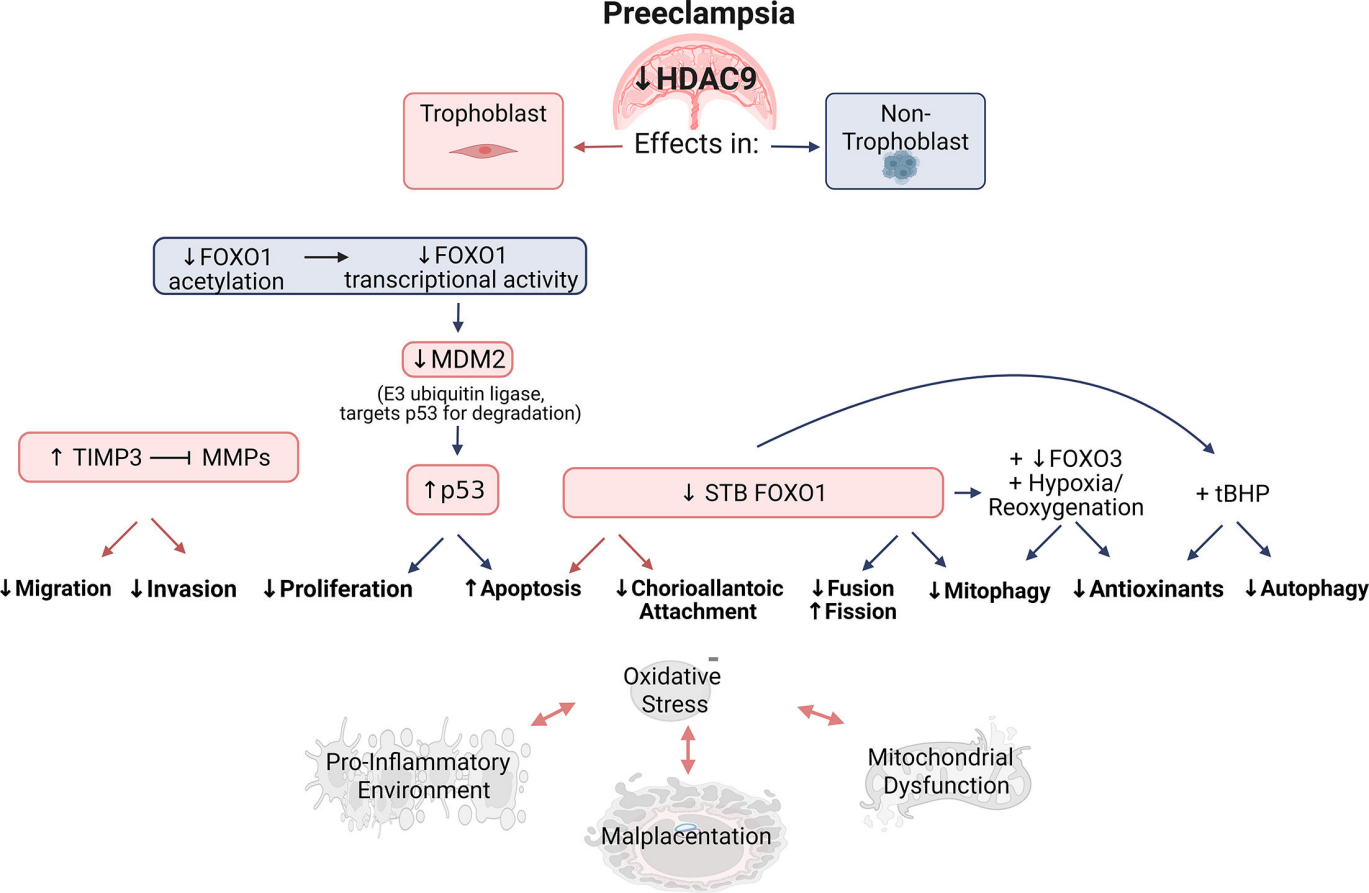
Established and proposed mechanisms of HDAC9-mediated regulation of placental function. Within trophoblast cells specifically, it has been established that decreased HDAC9 leads to increased *TIMP3* promoter activity, decreased MMP protein expression, and thus hindered migration and invasion, which are essential to proper placental development [40]. Research in non-trophoblast models indicates that HDAC9 facilitates FOXO1 acetylation and activity [51,115]; therefore, a lack of syncytiotrophoblast HDAC9 may be responsible for the decreased presence of syncytiotrophoblast FOXO1 in preeclampsia [116]. Embryonic deletion of *Foxo1* in mice resulted in increased apoptosis and failed chorioallantoic attachment [117]. In other systems, FOXO1 promotes maintenance of a healthy mitochondrial network, promoting fusion and mitophagy while reducing fission [118]. A loss of FOXO1 combined with other outlined stimuli relevant to preeclampsia (i.e. tBHP-induced oxidative stress, hypoxia/reoxygenation) also attenuated antioxidant capacity and impaired autophagy [119,120]. Further, HDAC9 knockdown in cancer cells increased apoptosis and diminished proliferation [60]. These phenotypes may be mediated via HDAC9-induced negative regulation of the tumor suppressor p53 [61], which is also reduced in preeclamptic placenta [121]. Diminished FOXO1 may also contribute to increased p53, as FOXO1 has been shown to promote transcription of MDM2, an E3 ubiquitin ligase that degrades p53 [122] and is also lower in preeclampsia [121]. Collectively, the illustrated cellular outcomes contribute to malplacentation and a tightly linked cycle of oxidative stress, mitochondrial dysfunction, and inflammation in preeclampsia [123,124]. Red boxes and red arrows denote mechanisms and cellular effects that have been specifically determined in trophoblast cells whereas blue boxes and blue arrows indicate speculation based on other cell types. ↑, increased; ↓, decreased; FOXO1 and FOXO3, forkhead box protein O1 and O3; HDAC9, histone deacetylase 9; MDM2, E3 ubiquitin-protein ligase Mdm2; MMPs, matrix metalloproteinases; p53, tumor protein 53; STB, syncytiotrophoblast; tBHP, tert-butyl hydroperoxide; TIMP3, tissue inhibitor of metalloproteinases 3.

Recent findings indicate placental HDAC9 is diminished in preeclampsia [34,40], and a loss of HDAC9 deters placental trophoblast cell migration and invasion via hyperacetylation of the tissue inhibitor of metalloproteinases 3 (*TIMP3*) promoter, increasing TIMP3 levels [40]. TIMP proteins oppose the migratory actions of matrix metalloproteinases (MMPs) and are anti-angiogenic [40,114]. Thus, it is probable that TIMPs promote inadequate spiral artery remodeling and insufficient oxygen extraction. However, knockdown of *TIMP3* following *HDAC9* downregulation only partially restores trophoblast invasion and migration, which supports the involvement of other targets as well [40] (Figure 2).

Beyond its control of *TIMP3*, HDAC9 has a complex relationship with the transcription factor myocyte enhancer factor 2 (MEF2) [90,125], a regulator of placental cell differentiation and invasion [126]. In the placenta, *MEF2* isoforms exhibit different expression patterns among human trophoblast subtypes (HTR8/SVneo cell line, extravillous-like; BeWo cell line, first trimester-like cytotrophoblast; and primary cytotrophoblasts). MEF2D was most prominent in the extravillous population but also expressed in cytotrophoblasts, with MEF2A being the highest abundance isoform in cytotrophoblasts [126]. Highlighting its functional significance, overexpression of MEF2D stimulated cytotrophoblast proliferation as well as HTR8/SVneo migration and invasion, and MEF2A promoted cytotrophoblast to syncytiotrophoblast differentiation. MEF2A expression was concomitant with p38 MAPK and ERK5 activation [126], both of which control a multitude of functions disrupted in preeclampsia, including cell division, differentiation, survival, angiogenesis, and immune responses [127–130]. In other systems, particularly skeletal muscle, MEF2 and HDAC9 participate in a complex feedback loop where MEF2 binds to the HDAC9 promoter and induces its expression. In turn, HDAC9 directly binds MEF2 and represses its transcriptional activity [125,131,132]. In the heart, HDAC9 represses the MEF2 transcriptional program indirectly by altering chromatin acetylation, a process regulated by cardiac stress-induced kinases [90]. These examples highlight HDAC9’s capacity to regulate MEF2 through distinct, context-specific mechanisms [90,125]. Given MEF2’s role in trophoblast function [126], disrupted HDAC9–MEF2 signaling may contribute to placental dysfunction in preeclampsia. HDAC9 loss could enhance MEF2 activity, serving as a compensatory response or a disease contributor, depending on other signaling pathways and timing. Further studies are needed to clarify HDAC9’s role in MEF2 regulation and the pathogenesis of this pregnancy disorder.

Further, HDAC9 may also facilitate placentogenesis via its relationship to hypoxia-inducible factor (HIF). HIF is a transcription factor often studied in the context of low oxygen tension during preeclampsia, but research indicates that HIF1 has oxygen-independent roles and communicates with HDACs to control trophoblast stem cell differentiation [133]. Deletion of *Arnt*, the heterodimeric partner of HIF1α, restricted trophoblast stem cell differentiation, decreased HDAC activity, and diminished HDAC9 nuclear localization in a 20% oxygen environment [133].

Translating pertinent information from non-trophoblast models, small interfering RNA against *HDAC9* attenuated gastric tumor growth by promoting apoptosis and limiting proliferation both *in vitro* and *in vivo* [60]. While this is efficacious for cancer treatment, it may be detrimental to placental development, where cytotrophoblast proliferation is essential for formation of the syncytiotrophoblast layer [60,134]. HDAC9 has been shown to associate with the proximal promoter of the tumor suppressor p53, inhibiting its transcription in osteosarcoma cells [61]. In preeclampsia, a condition marked by excessive trophoblast apoptosis [135,136], placental HDAC9 expression is reduced [34,40], and this aligns with elevated p53 protein levels observed in preeclamptic villous trophoblasts [121]. Consistent with these observations, HDAC9 knockdown in osteosarcoma cells suppressed proliferation and invasion, whereas overexpression encouraged these phenotypes [61], suggesting a similar role may exist in trophoblasts with diminished HDAC9 expression and elevated p53 in preeclampsia.

Further extrapolating its potential placental roles, HDAC9 may also exert indirect effects on the p53 pathway via modulation of the transcription factor forkhead box protein O1 (FOXO1). HDAC9-mediated deacetylation of FOXO1 has been linked to increased FOXO1 activity in human hepatoma cells [51,115]. FOXO1 is critical for structural development of the placenta in mice as its deletion leads to accelerated apoptosis and failed chorioallantoic attachment, likely through its regulation of vascular cell adhesion molecule [117]. Interestingly, FOXO1 has recently been shown to drive transcription of MDM2, an E3 ubiquitin ligase that targets p53 for degradation, which is proposed to promote cancer cell proliferation by dampening p53-mediated apoptosis [122]. This suggests a complex regulatory axis wherein FOXO1 activity, enhanced by HDAC9, could increase MDM2 expression and suppress p53. Contrarily, in preeclampsia, decreased FOXO1-positive nuclei in syncytiotrophoblasts [116], along with reduced MDM2 protein expression [121], may further amplify p53 signaling and exacerbate trophoblast apoptosis [122]. Thus, HDAC9 may promote placental health both by direct repression of p53 and by enhancing FOXO1 activity, leading to increased MDM2 expression and destabilization of p53 [40,42,51,61,116,121,122].

Within chondrocytes, knockdown of *FOXO1*, combined with exposure to the oxidizing agent tert-butyl hydroperoxide, led to decreased cell viability, autophagy-related proteins, and glutathione peroxidase 1 [119], an antioxidant protein also reduced in preeclamptic serum and placenta [137]. Additionally, mitochondria are a major source of reactive oxygen species which, in excess, create a vicious cycle of mtDNA mutations, mitochondrial damage, lipid peroxidation-induced cytotoxicity, apoptosis, and necrosis [138,139]. Both mitochondrial dysfunction and elevated oxidative stress are linked to the pathophysiology of preeclampsia [140–142] and generate a pro-inflammatory environment [123,124]. Since mitochondria are highly susceptible to damage and DNA mutations, a healthy mitochondrial network must be maintained through the careful coordination of biogenesis, fission, fusion, and mitophagy [143]. Mitochondrial biogenesis consists of the growth and synthesis of new mitochondria [144], whereas mitophagy refers to the selective degradation of dysfunctional mitochondria by autophagy [145]. Similarly, fission and fusion events control mitochondrial morphology and consist of the segregation and combination of mitochondrial contents, respectively [145,146]. Reanalysis of a placental microarray dataset (GSE75010) by our lab reveals that select markers of mitochondrial biogenesis (mitochondrial transcription factor A, *TFAM*; nuclear factor erythroid 2-related factor 2, *NFE2L2*) [147] and fusion (OPA1 mitochondrial dynamin-like GTPase, *OPA1*) [148] were decreased in preeclamptic samples. Contrarily, those for mitophagy (BCL2 interacting protein 3, *BNIP3*; BCL Interacting Protein 3 Like, *BNIP3L*) [149] and fission (dystrophin related protein 2, *DRP2*) [150] were increased. This is consistent with some other sources exploring similar markers [151,152], but overall the literature is divided [151–153].

Peroxisome proliferator-activated receptor γ coactivator 1α (PGC-1α), a master regulator of mitochondrial biogenesis [154], is positively regulated by FOXO1 in hepatic systems [51]. FOXO1 has also been shown to augment the expression of mitophagy and fusion-related proteins, while reducing those involved in fission, within both podocyte cells subjected to high glucose conditions and glomeruli from mice with diabetic nephropathy [118]. Although high glucose is not necessarily a stressor associated with preeclampsia, a high glucose environment stimulates the production of reactive oxygen species in a similar fashion to that experienced in preeclampsia, and glomerular damage is also evident in many women with this disorder [118,141,155–157]. Placental hypoxia is prevalent in some subtypes of preeclampsia [17]. Thus, more directly related, hypoxia/reoxygenation in rat neonatal cardiomyocytes resulted in decreased phosphorylated-FOXO1/FOXO1 (indicative of FOXO1 activation and nuclear localization) along with increased expression of antioxidant and mitophagy-related proteins [120]. The loss of cardiomyocyte *Foxo* (both *Foxo1* and *Foxo3*) within a whole-animal model led to a worsening of cardiac function, oxidative stress, decreased expression of mitophagy and antioxidant proteins, and increased apoptosis following myocardial infarction [120]. However, there is some conflicting evidence pertaining to the protective role of FOXO1 following ischemia-related injuries that may be due to tissue and cell-specific differences. For instance, administration of a FOXO1 inhibitor for several days prior to renal ischemia/reperfusion injury instead ameliorated the generation of mitochondrial reactive oxygen species, mitochondrial-induced apoptosis, and tubular damage [158]. Thus, abolishing the effects of FOXO1 within the kidney may be protective in certain pathological states [158]. Nonetheless, considering FOXO1 is essential for proper placental development [117] and the presence of hypoxic stress in preeclampsia [17], global FOXO1 inhibition would likely be detrimental during pregnancy. The facilitation of FOXO1 via HDAC9 may be critical to maintaining mitochondrial integrity and cellular viability under placental stress.

Taken together with its interactions across multiple pathways, these data foster the speculation that HDAC9 has a multitude of molecular targets essential to proper placental patterning and function. Its involvement in control of TIMP3 [40], MEF2 [125], HIF [133], FOXO1 [51,115], and p53 [61] underscores the complexity of HDAC9’s actions, and understanding its regulation and downstream effects in this gestational syndrome may reveal key mechanistic insight regarding the pathogenesis of the disorder.

## Modulation of immunity

**Figure 3:**
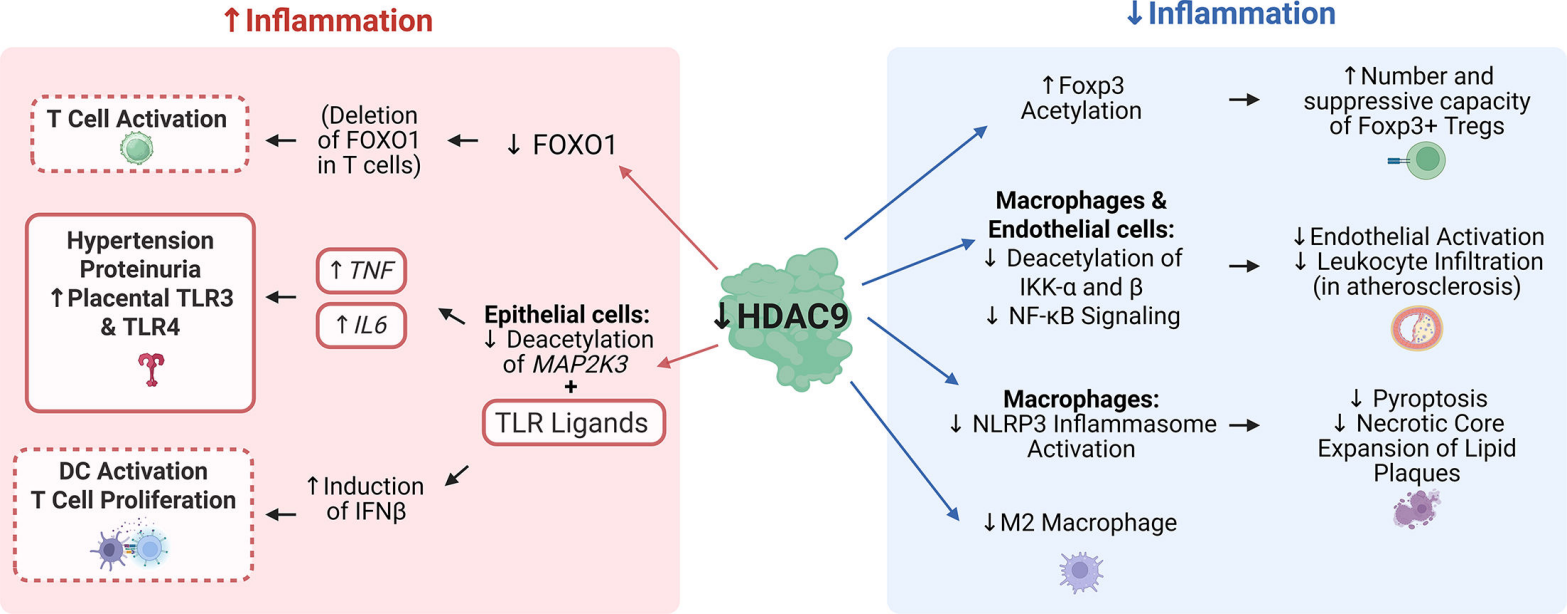
Pro- vs. anti-inflammatory effects of HDAC9. The specific role of HDAC9 as it relates to immunity and inflammation has yet to be determined in the context of preeclampsia, and other models indicate the loss of HDAC9 may have pro- (shown in the pink box) [166,167] or anti-inflammatory (shown in the blue box) consequences [163–165,169]. Thus far, it has been determined that HDAC9 is decreased in preeclamptic syncytiotrophoblasts, which are a type of epithelial cell [40]. Evidence from other epithelial cells, specifically keratinocytes, suggests a lack of HDAC9 would have pro-inflammatory consequences [166,167]. Further, HDAC9’s control over FOXO1, combined with known effects of FOXO1 deletion in T cells, also suggests attenuated HDAC9 would perpetuate immune activation [170]. For these studies, molecular mediators and downstream cellular effects that directly coincide with those observed in preeclampsia are highlighted in pink boxes, whereas responses that closely resemble preeclampsia are surrounded by a dashed pink line. However, there is also an opposing field of research suggesting removal of HDAC9 would be anti-inflammatory, particularly in maternal immune cells. In a mouse model of colitis, HDAC9 deletion enhanced Foxp3^+^ Treg number and suppressive capacity [168], but preeclampsia is associated with reduced Treg abundance [169]. HDAC9 deficiency has also been shown to promote anti-inflammatory M2 macrophage polarization in the uterus [169], in contrast to the M1 dominance seen in preeclampsia [171]. In vascular disease, HDAC9 promotes NF-κB and NLRP3 inflammasome activation [163,164], both of which are upregulated in preeclamptic placentas [172–175]. These findings highlight that HDAC9’s inflammatory role is likely context- and cell-type dependent, underscoring the need for further investigation in pregnancy-specific models. DC, dendritic cell; FOXO1, forkhead box protein O1; Foxp3, forkhead box protein P3; HDAC9, histone deacetylase 9; IFNβ, interferon beta; IKK-α/β, inhibitor of kappa B kinase alpha/beta; IL6, interleukin 6; MAP2K3, mitogen-activated protein kinase kinase 3; NF-κB, nuclear factor kappa-light-chain-enhancer of activated B cells; NLRP3, NOD-like receptor family pyrin domain containing 3; TLR, toll like receptor; TNF, tumor necrosis factor; Treg, regulatory T cells.

Pregnancy is maintained by an intricate balance of pro- and anti-inflammatory mediators, as the maternal immune system encounters paternal antigens via its interactions with the fetoplacental unit [159]. At the same time, trophoblasts and maternal immune cells are exposed to pathogen or damage-associated molecular patterns (PAMPs/DAMPs) from stressed or dying cells, injury, and pathogens that trigger responses from both cell types [160]. Thus, immunological adaptations occur to promote a tolerant milieu of the semi-allogenic fetus while preserving the capacity for immune defenses [161,162], and preeclampsia is accompanied by dysregulated immune activation and heightened inflammation [78]. Although the role of HDAC9 in modulating immunity and inflammation has not been defined in pregnancy or preeclampsia, evidence from related models suggests that HDAC9 primarily regulates innate immune responses [163–167], with limited evidence pointing to potential effects on adaptive immunity [168]. Interestingly, HDAC9 is considered anti-inflammatory in some contexts [166,167] and pro-inflammatory in others [163–165,168]. This segment explores the dual role of HDAC9 and implications for this gestational disorder (Figure 3).

### Anti-inflammatory effects of HDAC9

Briefly, the innate immune system initiates early protection against potential threats through receptors such as toll-like receptors (TLRs) that recognize PAMPs or DAMPs released during cellular stress. These include cell-free DNA, RNA, mitochondrial DNA (mtDNA), membrane components, and other stress-related molecules [176,177]. TLRs 1–9 are expressed in the placenta as well as decidual macrophages and natural killer cells [178–180]. Within the placenta, these receptors were localized to cytotrophoblasts, syncytiotrophoblasts, endothelial cells, and Hofbauer cells, with distinct temporal and spatial patterns across gestation [181–183]. Their activation during pregnancy promotes immune cell recruitment and cytokine secretion [176], with different cell types displaying different patterns of cytokine release [180]. Placental TLR3 [178], TLR4 [184], and TLR9 [185] are increased in preeclampsia. Animal and cell culture models confirm that activation of these receptors recapitulates changes concomitant with the disorder, including elevated systolic blood pressure [186], proteinuria [186], reduced trophoblast migration [185,187], spiral artery defects [188], and alterations in angiogenic factors [185].

Linking TLRs to HDAC9, studies have identified HDAC9 as an important regulator of immune responses to microbial cues, promoting immune tolerance in the skin [166,167]. Like these barrier sites, the fetal-placental unit is exposed to microbes and their metabolites throughout pregnancy [189], which can regulate HDACs and shape immune responses to these external signals. HDAC8 and HDAC9 impede the inflammatory effects of TLR stimulation in keratinocytes through deacetylation of mitogen-activated protein kinase kinase 3 (*MAP2K3*), a regulator of cytokine production. More specifically, separately silencing these HDACs promoted a more severe induction of interferon beta to TLR ligands, which promotes dendritic cell activation and T cell proliferation [167]. Further, the administration of polyionosinic-polycytidylic acid [167], a double-stranded RNA mimetic recognized by TLR3 [190], increased tumor necrosis factor (*TNF*) and interleukin 6 (*IL6*) mRNA [167], which was exacerbated by HDAC inhibition [167]. Similarly, microbial short-chain fatty acids (SCFAs), produced by *Propionibacterium acnes* under lipid-rich, low-oxygen conditions, can inhibit HDAC8 and HDAC9 in keratinocytes, leading to heightened cytokine production in response to TLR2 and TLR3 stimulation. Sodium butyrate, an SCFA produced by microbes and an HDAC9 inhibitor, increased IL-6, TNFα, interleukin 8 (IL8), and chemokine ligand 2 (CCL2) in response to a TLR2/6 ligand but had the opposite effect in monocytes [166]. This study highlights how specific microbial metabolites can epigenetically break immune tolerance in some tissues, further emphasizing the context-specific role of HDAC9 in modulating local immune responses to microbial-derived signals.

Consistent with these investigations in skin, SCFAs support placental growth and vascularization in mice [191]. Moreover, amniotic fluid contains skin commensals such as *Propionibacterium acnes [189]*, suggesting microbiota-derived metabolites may shape the intrauterine environment. Nuclear staining for HDAC9 protein is decreased in preeclamptic syncytiotrophoblasts [40], the outer villous layer directly exposed to maternal circulating factors [26], and its loss may perpetuate the inflammatory signaling present within this tissue. Of the aforementioned immune effectors, plasma levels, placental immunostaining, and placental mRNA for IL6 and TNFα were elevated in patients with preeclampsia at both earlier (28–36 weeks) and later (> 37 weeks) gestational ages [192,193]. Decidual IL-6 also increased at 29.7 ± 1.2 weeks and was stimulated by TNFα in leukocyte-free first trimester decidual cell culture [194]. In pregnant mice, TNFα infusion provoked maternal hypertension, proteinuria, and upregulated placental TLR3 and TLR4 [195]. Relating to the dendritic-induced T cell proliferation aspect [167], preeclampsia is characterized by an enhanced presence of decidual dendritic cells [196]. Dendritic cells derived from women with preeclampsia also exhibited higher cluster of differentiation (CD)83, CD80, and CD86 [197], denoting mature dendritic cells capable of activating naïve T cells [196,197]. The preeclamptic population of dendritic cells also secreted more IL-23, which acts in concert with other interleukins to promote CD4+T cell differentiation into the Th1 and Th17 subtypes [197]. Th1 and Th17 cells are generally associated with allograft rejection [198] and are the dominant subset present in preeclampsia [199,200], whereas the more tolerant Th2-induced cytokine responses are diminished [198,200].

Further, HDAC9 may be indirectly related to immunity and inflammation through its control of FOXO1 [51,170], which is largely associated with resistance to oxidative stress and immune tolerance, among its many roles [116,201]. As mentioned, HDAC9 alters the acetylation status of FOXO1, promoting its activation [51]; therefore, some deleterious cellular complications in preeclampsia may be a consequence of FOXO1 dysregulation secondary to the loss of HDAC9. T cell-specific *Foxo1* depletion in mice revealed that *Foxo1* is necessary for the preservation of naïve T cells, and its elimination leads to T cell activation and differentiation [170]. This aligns with the shift toward a more pro-inflammatory, differentiated T cell profile observed in preeclampsia [202].

### Potential pro-inflammatory role in the maternal compartment

In contrast with the previous immune studies, a body of literature supports a pro-inflammatory role of HDAC9 [163–165,168]. HDAC9 may impose differential effects in maternal and fetal compartments, including the placenta, due to differences in genetic makeup and microenvironment. However, pregnancy and its disorders are accompanied by unique hormonal and physiological adaptations that may regulate HDAC9 itself [88,100] (including changes in angiotensin II and estrogen, which are discussed in the subsection titled *GPCR Associated Kinases and Oxidative Stress as Potential Drivers of HDAC9 Dysregulation in Preeclampsia*) or affect the targets it acts upon. Thus, while these other states are informative, more work needs to be done specifically in the context of different cell types and tissue in pregnancy and preeclampsia.

Highlighting its potential inflammatory effects in maternal immune cells, HDAC9 contributes to inflammatory bowel disease by suppressing regulatory T cell (Treg) function. Using a mouse model of colitis, researchers showed that HDAC9 knockout increases both the number and suppressive capacity of Foxp3+Tregs, partly by preserving Foxp3 acetylation status [168]. Contrarily, preeclamptic individuals have a lower number of Treg cells [169], and depletion in early pregnancy within mice elicits key features, including exacerbated inflammatory signaling and increased uterine artery vasoactivity [203].

HDAC9 also appears to shape macrophage polarization early in gestation, which is essential to maintaining pregnancy [165]. In the uterus, attenuating HDAC9 shifts macrophage differentiation toward the anti-inflammatory M2 phenotype [165]. Preeclampsia is generally associated with an increased abundance of pro-inflammatory M1 macrophages in the decidua at term [171], suggesting a shift away from the M2-dominated environment required for immune tolerance and vascular remodeling [204]. While HDAC9 is down-regulated in syncytiotrophoblasts during preeclampsia, its role in decidual macrophages over the course of gestation remains unclear and warrants further investigation.

Genome-wide association studies have linked risk alleles that increase HDAC9 expression to atherosclerosis, and emerging research has identified nuclear factor kappa-light-chain-enhancer of activated B cells (NF-κB) and the NOD-like receptor family pyrin domain containing 3 (NLRP3) inflammasome as key downstream targets of HDAC9 in the regulation of vascular inflammation [163,164]. NF-κB is a well-known transcription factor that governs inflammatory responses [172], and it is markedly upregulated in preeclamptic syncytiotrophoblasts but also cytotrophoblasts, the villous core, leukocytes within placental vessels [172] as well as maternal serum [173]. NF-κB has an extensive role in the placenta, contributing to trophoblast migration and angiogenesis in physiological pregnancy, while also modulating factors that promote oxidative stress, inflammation, and vascular dysfunction in preeclampsia [174]. In vascular disease, HDAC9 promotes atherosclerotic plaque vulnerability through the deacetylation of inhibitory kappa B kinase (IKK)-α and β, ultimately leading to increased NF-κB-mediated transcription of pro-inflammatory chemokines, cytokines, or adhesion molecules in macrophages and endothelial cells [163]. Furthermore, inhibiting HDAC9 via TMP195, a class IIa inhibitor, limited lesion progression in a mouse model of atherogenesis, which was attributed to attenuated endothelial activation, reduced leukocyte infiltration, and curbed NF-κB target gene expression [163]. Building upon this finding, the NLRP3 inflammasome is a multiprotein complex that activates caspase-1, promoting the maturation and secretion of pro-inflammatory cytokines such as IL-1β and IL-18 and triggering pyroptosis, a form of programmed inflammatory cell death [164,175]. NF-κB signaling acts as a critical first step in activating the NLRP3 inflammasome by inducing the expression of key components such as NLRP3 itself and pro-inflammatory cytokines like pro-IL-1β, thereby priming immune cells for inflammasome assembly [205]. However, in macrophages, HDAC9 promotes NLRP3 inflammasome activation via direct deacetylation, thereby facilitating its assembly and enhancing its functional activity independent of NF-κB-mediated transcriptional priming [164]. This promotes pyroptosis and contributes to necrotic core expansion in lipid plaques. Although the specific cell types mediating the pro-atherogenic effects of HDAC9 remain incompletely defined, deletion of a cis-regulatory element within the HDAC9 locus increased HDAC9 expression, specifically in myeloid cells and bone marrow-derived macrophages, without affecting expression in T cells, smooth muscle cells, or endothelial cells. Importantly, this regulatory element overlaps with a genomic region that contains common variants identified by genome-wide association studies as risk factors for cardiovascular disease [164].

These findings in vascular disease raise the possibility that similar mechanisms may operate in preeclampsia, a disorder similarly characterized by endothelial dysfunction, exaggerated innate immune activation, and impaired vascular remodeling [76,78]. Both NF-κB and NLRP3 inflammasome signaling are upregulated in preeclamptic placenta and maternal circulation, where they may contribute to increased cytokine production, oxidative stress, and systemic inflammation [172–175]. Stressed cells, including syncytiotrophoblasts, in preeclampsia are known to shed necrotic debris, extracellular vesicles, DNA, and other danger signals which can act as damage-associated molecular patterns (DAMPs) that trigger inflammasome activation in maternal immune cells [175]. Given that HDAC9 promotes both NF-κB and NLRP3 inflammasome activation in macrophages [163,164], its dysregulation may amplify these inflammatory cascades in preeclampsia. Moreover, the suspected contrasting patterns of HDAC9 expression (i.e. reduced in syncytiotrophoblasts but potentially increased in maternal immune cells) highlight a spatial disconnect that may contribute to the inflammatory imbalance characteristic of the disorder.

## Role in angiogenesis

Preeclampsia is attributed to an imbalance of placental-derived circulating pro-angiogenic (vascular endothelial growth factor, VEGF; placental growth factor, PlGF; membrane-bound endoglin, ENG) and anti-angiogenic (soluble fms-like tyrosine kinase 1, sFLT1; soluble endoglin, sENG) [30,70] factors. VEGF and its counterpart PlGF are present in trophoblast and perivascular cells to stimulate de novo vasculogenesis as well as vessel expansion via endothelial sprouting, or angiogenesis [206,207], but these ligands are sequestered by sFLT1. Though not as directly involved in vascular remodeling, endoglin is located on endothelial cells, syncytiotrophoblasts, and early columnar cytotrophoblasts and enhances angiogenesis through its control of cell proliferation [208,209]**.** Similarly, its soluble version, sENG, opposes endothelial tube development [210]**.** To some degree, the angiogenic defects in preeclampsia may be ascribed to reductions in HDACs, including HDAC9. Pan HDAC inhibition impaired VEGF-induced angiogenic activity in human umbilical vein endothelial cells (HUVECs), leading to upregulation of the VEGF competitor semaphorin III, and mitigated capillary sprouting from rat aortic rings [211]. Moreover, manipulations of *HDAC9* in both HUVECs and animal models confirmed that HDAC9 supports vessel formation, patterning, and perfusion, in part by repressing the microRNA (miR)-17*–92* cluster [212]. Reinforcing the relevance of miR-17–92 in preeclampsia, Wang et al. found members of the miR-17–92 cluster, namely miR-17 and miR-20a, were upregulated in preeclamptic placenta [213]. Predicted targets included *HIF1A*, *VEGFA*, and *EFNB2* [213], with EFNB2 being known to promote endothelial sprouting by facilitating the internalization and downstream signaling of VEGFR2 and VEGFR3 [**214,215**].

Hypoxia-inducible factors (HIFs) traditionally respond to low oxygen tension by potentiating transcription of angiogenic factors, including VEGF, PlGF, and ENG [216–219], but HDAC9 may be necessary for this to occur. This is evident as small interfering RNA against *HDAC9* hindered the translation of HIF1α [38] although the nuclear translocation of HIF1α was not explored. Related to these findings, the administration of a pan-HDAC inhibitor, vorinostat, reduced nuclear to cytoplasmic HIF1/2α levels and gene expression of *VEGFA* in tumorigenic cell lines [39]. While hypoxia can promote the release of angiogenic factors, it can also increase sFLT1 production via the c-Jun NH2 -terminal kinase/activator protein-1 (JNK/AP-1) pathway [220]. However, the cellular effects of hypoxia are complex and vary within different subsets of placental cells. Decreased environmental oxygen mediates the release of sFLT1 in cytotrophoblasts, which was further exacerbated by differentiation into syncytiotrophoblasts [221,222], but this response was not detected in villous fibroblasts or endothelial cells [221]. Similarly, in placental tissue from severe early-onset cases of preeclampsia, sFLT1 mRNA and protein levels were increased relative to preterm control samples [223]. sFLT1 was primarily located within vascular regions, the syncytiotrophoblast layer, and syncytial knots in preeclamptic tissue [223]. This regional distribution may relate to the secretory role of syncytiotrophoblasts, along with their inherent vulnerability to oxidative damage and generalized cell stress throughout pregnancy [26,224]. Hypoxia facilitates the generation of mitochondrial-derived intracellular reactive oxygen species in BeWo cells [225], which resemble villous cytotrophoblasts and are capable of fusing into syncytial-like structures [226], and this response was attenuated by administration of a mitochondrial-targeted antioxidant [225]. Linking hypoxia, sFLT1, and markers of oxidative stress, exposing placental villi to 2% oxygen resulted in increased production of sFLT1, VEGF, and lipid peroxidation [227], an oxidative reaction associated with tissue damage [228], and decreased PlGF [227]. As far as whole-animal physiology, treatment with recombinant sFLT1 in pregnant rats elevated blood pressure, impaired vasorelaxation, lowered the abundance of free VEGF, and increased superoxide production, but vasoactive function was restored by a superoxide scavenger [229]. Together, these findings suggest that the pathogenesis of preeclampsia involves a convergence of hypoxia-induced oxidative stress, dysregulated HDAC-mediated control of angiogenic signaling, and an imbalance of pro- and anti-angiogenic substances that may be partially relieved by enhanced oxidant defense systems.

## Relationship to G-protein-coupled receptor (GPCR) hormones, regulators, and signaling intermediates in Preeclampsia

### GPCR hormones in pregnancy and preeclampsia

Pregnancy is marked by cardiovascular adaptations, including increased plasma volume, cardiac output, and vasodilation, to promote adequate uteroplacental perfusion and meet the metabolic demands of gestation [230,231]. These changes are orchestrated by a complex interplay of maternal and placental hormones including estrogen, progesterone, relaxin, and human chorionic gonadotropin, which promote vasodilation and fluid retention through both genomic and nongenomic mechanisms [232–238]. A pregnancy-specific shift in osmoregulation lowers the threshold for vasopressin release, facilitating renal water reabsorption, thirst, and expansion of extracellular fluid volume [239–242]. Circulating levels of angiotensin II are also elevated, likely in response to reduced systemic resistance and renin-angiotensin system activation. Yet, their vasoconstrictive effects are attenuated, a phenomenon attributed in part to increased levels of vasodilatory mediators such as estrogen, progesterone, nitric oxide, and prostacyclin [236,243,244].

**Figure 4:**
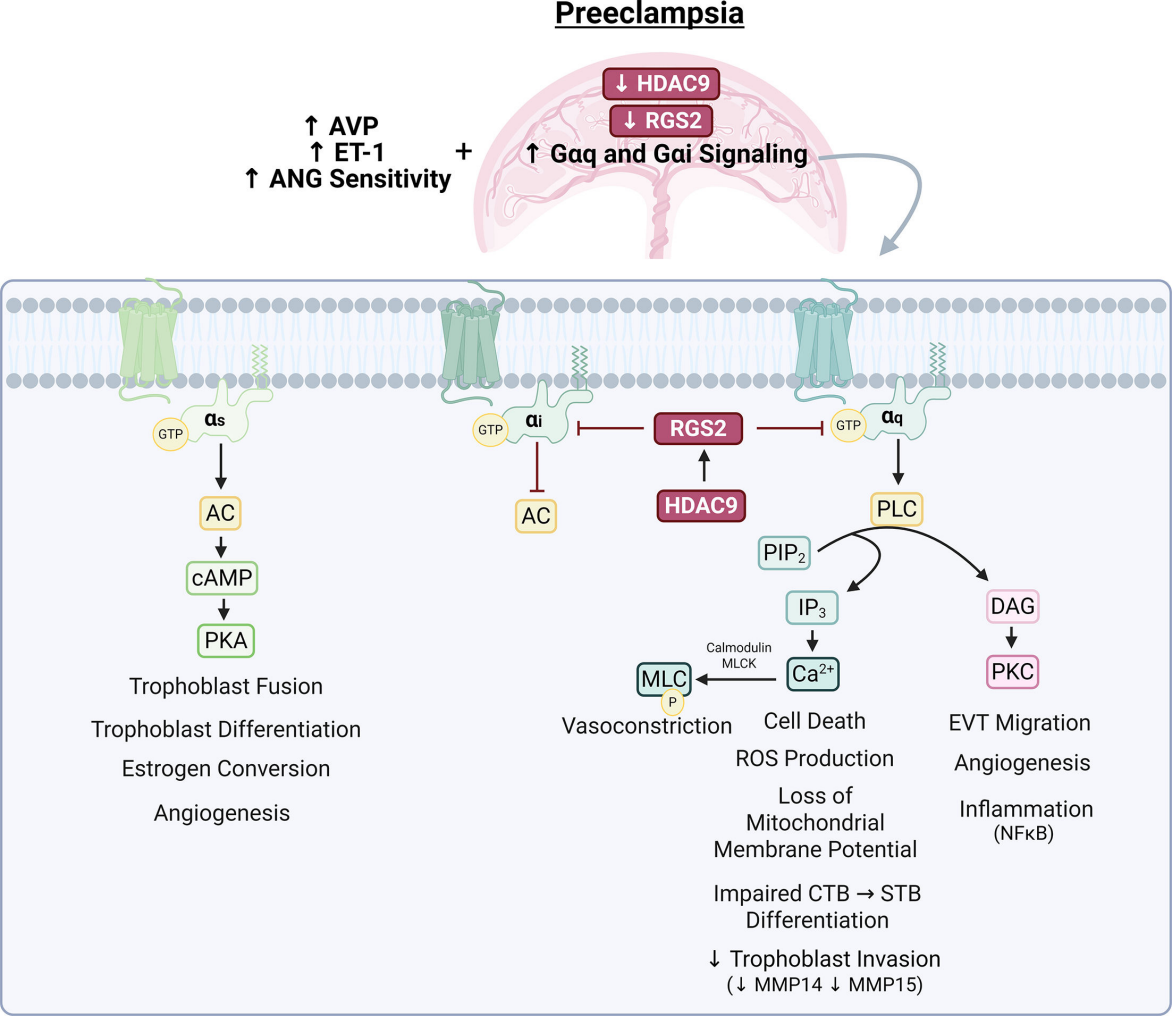
Effects of G-protein-coupled receptor cascades in the placenta. Preeclampsia is attributed to increased placental GPCR signaling in response to the pressure-regulating hormones AVP, ET-1, and ANG, which activate various pathways and thus elicit different cellular responses depending on the specific G-protein present [35,37,102,246]. There are several Gα subtypes, including Gαs, Gαi, and Gαq. Their corresponding receptors, classical cellular cascades, and downstream placental effects have been detailed in the figure [247,248,255–271]. RGS2 is an important buffer of excess Gαq and Gαi signaling [34,272–274] and is reduced in preeclampsia, which may promote overall placental dysfunction and stress [34,254,275]. Relating these cascades to HDACs, there is an established transcriptional dependence of *RGS2* on HDAC9 in human trophoblast cells [34]; therefore, the lack of placental HDAC9 in preeclampsia likely contributes to the detrimental cellular consequences of elevated Gαq and Gαi input from AVP, ET-1, and ANG. AC, adenylyl cyclase; ANG, angiotensin II; AVP, vasopressin; Ca^2+^, calcium; cAMP, cyclic adenosine monophosphate; CTB, cytotrophoblast; DAG, diacyl glycerol; ET-1, endothelin-1; GTP, guanosine triphosphate; HDAC9, histone deacetylase 9; IP_3_, inositol trisphosphate; MLC, myosin light chain; MLCK, myosin light chain kinase; MMP14 and 15, matrix metalloproteinase 14 and 15; NF-κB, nuclear factor kappa-light-chain-enhancer of activated B cells; P, phosphate; PIP_2_, phosphatidylinositol 4,5-bisophosphate; PKA, protein kinase A; PKC, protein kinase C; PLC, phospholipase C; RGS2, regulator of G-protein signaling 2; STB, syncytiotrophoblast.

However, women with preeclampsia display heightened sensitivity to or secretion of angiotensin II [33] and vasopressin [37], which are known to facilitate the release of endothelin-1 [245], as well as decreased nitric oxide-dependent vasodilation [246]. Angiotensin II, vasopressin, and endothelin-1 all signal through G-protein-coupled receptors (GPCRs) [247,248]. These ligands are produced both systemically and locally by maternal tissues [239,249,250]; however, the placenta also expresses receptors and enzymes for their synthesis [35,67,103,251], serving as both a target and amplifier of these pathways. While GPCR signaling does have essential physiological functions [252], aberrant activation of these cascades can result in a state of vasoconstriction [32,33,253]**,** abnormal placental development [35,36,254], and trophoblast stress [36]. HDAC9 has emerged as an underlying contributor via its control of regulator of G-protein signaling (RGS) proteins that dampen GPCR activation [34,254], and GPCR kinases may also affect the localization of HDAC9 [88,100], creating an amplified feedback loop that reinforces excessive GPCR signaling in preeclampsia (Figure 4).

### Placental effects of GPCR pathways

GPCR signaling occurs through four main pathways (Gαq, Gαi, Gαs, Gα12/13) depending on the specific α subunit coupled to the receptor [255], and these variations contribute to the diversity of responses to GPCR hormones. Considering their multitude of functions and potential crosstalk, the role of each signaling cascade has yet to be completely dissected. However, disinhibition of these pathways has been clearly linked to abnormal placental development and preeclamptic phenotypes [34,36].

Angiotensin II, vasopressin, and endothelin-1 are traditionally linked to blood pressure regulation but also have receptors present in trophoblast cells [81,83,251,276–278], and exert effects including increased sFLT1 [279], diminished trophoblast invasion [256,280], and impaired syncytiotrophoblast differentiation [281]. Many of their receptors predominantly couple to the Gαq cascade (i.e. AT1, V1A, and in some instances ETA and ETB) [247,248,282,283], which mediates its downstream effects through protein kinase C (PKC) and calcium liberation [247,248]. PKC coordinates extravillous trophoblast migration, angiogenesis, and inflammation through its interactions with scaffolding proteins [257,258], as well as through its downstream effects on VEGF (see previous section: *Role in Angiogenesis*) [284], and NF-κB signaling (see previous subsection: *Potential Pro-Inflammatory Role in the Maternal Compartment*) [258,285], the latter of which is dramatically upregulated in preeclamptic placentas [172]. Intracellular calcium evokes vascular smooth muscle contraction by binding with calmodulin to activate myosin light chain kinase (MLCK). This renders MLCK capable of inducing myosin light chain (MLC) phosphorylation, allows actin–myosin cross-bridge interaction, and thereby promotes increased vasomotor tone [248,259]. Likewise, excess calcium in placental explants is associated with production of reactive oxygen species, disrupted mitochondrial membrane potential, cell death, and impaired cytotrophoblast to syncytiotrophoblast differentiation [260]. In this process of differentiation, cytotrophoblasts fuse to form multinucleated syncytiotrophoblasts with endocrine function [286,287]. Though HDAC9 has not been directly linked to this process, HDAC9 is localized to the syncytiotrophoblast layer in healthy placentas and is largely absent in preeclampsia [40]. A lack of HDAC9 prevents transcription of regulator of G-protein signaling 2 (*RGS2*) in human trophoblast cells, and RGS2 is a prominent inhibitor of the Gαq pathway [34,272,273]. Further, we recently reported evidence of elevated Gαq signaling in preeclamptic syncytiotrophoblasts, and selective activation of Gαq within this cell type in mice was sufficient to induce features of the disorder via a mechanism dependent on mitochondrial-derived reactive oxygen species [36].

While there are currently a lack of early biomarkers for the identification of preeclampsia, our lab discovered that plasma copeptin, the pro-segment of vasopressin, is elevated in preeclamptic individuals prior to the manifestation of other symptoms [37]. Follow-up experiments indicated a causal relationship between these two parameters such that vasopressin infusion in mice elicited features of preeclampsia including diminished spiral artery diameter, placental oxidative stress, and maternal end organ damage [35]. *In silico* reanalysis of GSE75010 confirmed that the gene encoding vasopressin (*AVP*) and its Gαq-coupled vasopressin 1 A receptor (*AVPR1A*) [261] were increased in preeclamptic placentas—other vasopressin receptors were present but not significantly changed in the disorder [35]. Further, our vasopressin infusion mouse model also showed placental transcriptomic enrichment of calcium signaling and inositol phosphate compound pathways, supporting a prominent Gαq-mediated effect, despite potential activation of other cascades [35]. The role of HDAC9 in this specific situation has yet to be identified. However, HDAC9 is present in vasopressin neurons [288], and HDAC inhibition increases vasopressin immunoreactivity [289]. Thus, at some level, HDAC9 may be a culprit of elevated vasopressin secretion or signaling, but there is not currently any direct evidence to support this notion.

G-protein signaling following endothelin-1 stimulation is notoriously heterogenous, and endothelin-1 receptors appear to act through several pathways [262]. However, studies suggest that endothelin B receptors (ETB) within trophoblasts couple to the Gαq cascade [290]. Treatment of first trimester villous placental explants with endothelin-1 and selective receptor antagonists revealed that ETB-mediated responses attenuate MMP14 and MMP15 expression as well as trophoblast invasion [256]. Other MMPs (i.e. MMP2 and MMP9) have gestational-age-dependent roles and foster spiral artery remodeling in early pregnancy but later perpetuate aberrant release of vasoactive substances [291]. Class II HDACs have been shown to promote MMP9 expression in primary amnion cells [292], but the specific relationship between HDAC9 and other MMPs in preeclampsia has yet to be elucidated.

Gαi and Gαs also regulate maternal physiology and the fetoplacental interface during pregnancy [263–266,293,294]. These two subunits act in opposition such that Gαs promotes adenylyl cyclase (AC) mediated generation of cyclic adenosine monophosphate (cAMP), and thus protein kinase A (PKA) activation, whereas Gαi inhibits AC [267]. PKA has a diverse set of roles in the placenta and stimulates trophoblast fusion, differentiation, estrogen conversion, progesterone synthesis, and the release of angiogenic factors including PlGF and VEGF [263–266,268–270,295]. Angiotensin II type 2 receptors (AT2R) are localized to vascular smooth muscle cells within the umbilical cord and placenta [296] and generally signal through Gαi subunits [297]. Unlike its more ‘pathogenic-associated’ counterpart (i.e. angiotensin II type 1 receptor), signaling via the AT2R tends to be recognized as ‘favorable’, and this receptor increases uteroplacental blood flow to allow greater oxygen extraction [298]. HDAC9 does not have an obvious role in the control of this particular GPCR pathway in preeclampsia except for its implications in the regulation of *RGS2*, which can also act on Gαi [274].

 Collectively, these findings support the involvement of various GPCRs in a multitude of placental and vascular events, and the interplay among each GPCR cascade and its downstream effects creates a complex system that is difficult to unravel. However, it is evident that abnormal GPCR signaling directly relates to the constellation of symptoms present in preeclampsia [34–36,249,256,299]. The link between GPCR activation and attenuated HDAC9 in preeclampsia is further discussed in the subsection titled *GPCR-Associated Kinases and Oxidative Stress as Potential Drivers of HDAC9 Dysregulation in Preeclampsia*.

### Disinhibition of GPCR signaling in preeclampsia via HDAC9-mediated control of regulator of G-protein signaling (RGS) proteins

RGS proteins dampen the strength and duration of GPCR activation [300] and thus have a role in offsetting pregnancy-associated increases in blood pressure. More specifically, RGS proteins hydrolyze the GTP bound to an active Gα subunit, causing it to re-associate with the βγ heterodimer, halting the downstream effects of both [301]. However, in preeclampsia and other disease states, RGS proteins are insufficient to terminate this signaling [244]. Among the large family of RGS proteins, RGS2 and RGS5 are the two that have been most studied in the context of this disorder [34,302–304]. These two proteins are key attenuators of GPCR signaling in cardiac, smooth muscle, placental, renal, and neural tissues [34,244,305,306], and RGS proteins exhibit selectivity for specific G-protein subtypes [274]. It has previously been determined that RGS2 and RGS5 are key Gαq and Gαi attenuators, with RGS2 eliciting a greater specificity for Gαq [244,272–274]; however, recent reports indicate RGS2 is relatively versatile and is also a potent controller of the lesser-studied Gα15 and Gαo subunits, but not Gα12/13 [274].

The dysregulation of RGS proteins has been highly explored in the vasculature [244,273,307], and indeed, *RGS5* mRNA levels are lower in myometrial arteries from preeclamptic women [303]. Furthermore, a single nucleotide polymorphism in the *RGS2* 3’ untranslated region, termed rs4606, is correlated with an increased risk for the development of preeclampsia in overweight women [304,308]. This particular SNP was more common in women who also displayed acute atherosclerosis in decidual spiral arteries [304], reinforcing the link between this disorder and vascular function. In mice, homozygous knockout of *Rgs5* elicited hypertension that was exacerbated during pregnancy, whereas mice with heterozygous disruption were not challenged with hypertension until pregnant [303]. These effects were due to an increased responsiveness to angiotensin II and oxidative stress [303], both relevant aspects of human preeclampsia [253]. Likewise, *Rgs2* and *Rgs5* are necessary for maintaining uterine blood flow in mice, and a lack of *Rgs2* elevates resistive index [302]. Although the precise effects of HDACs on RGS2 and RGS5 have not been explored in the vasculature, siRNA-mediated knockdown of HDAC9 and pan-HDAC inhibition both suppressed *RGS2* in human trophoblast cells [34]. Pan-HDAC inhibition also decreased *RGS5* in neural progenitor cells [309], suggesting there may be an important relationship between these modulators in preeclamptic women.

In addition to the vascular implications [244,273,307], our lab recently made the novel discovery that *RGS2* mRNA is decreased in the human preeclamptic placenta [34]. Heterozygous knockout in the fetoplacental unit of mice caused key characteristics of the disorder including diastolic hypertension and proteinuria, suggesting a causative relationship between the loss of *Rgs2* and preeclampsia symptoms [34]. *Rgs2*-deficient mice had attenuated placental vascularization, despite a lack of differences in spiral artery morphology or expression of canonical hypoxia-related genes. This finding illustrates the complexity of this disorder and suggests that hypoxia and exacerbated GPCR signaling may independently elicit features of preeclampsia [34]. Mechanistic studies confirmed that, like in other tissues, *RGS2* is a CREB target gene in human trophoblast cells. However, despite increases in pCREB/totalCREB in preeclamptic placenta, CREB occupancy at the *RGS2* promoter was inhibited. Distinct from other canonical CREB target genes, *RGS2* expression was dependent on *HDAC9*, which was also lower in human preeclamptic placenta [34]. The decline in both *HDAC9* and *RGS2* suggests a non-traditional role for HDAC9 in the control of *RGS2* where it acts as an activator rather than a repressor.

The mechanism by which HDAC9-mediated dysregulation of *RGS2* occurs remains unknown. Data in PC-12 cells indicate that HDACs are necessary for recruitment of RNA polymerase II and transcription factor II B during preinitiation complex assembly in a subset of CREB genes [49], but this has yet to be explored in relation to RGS2. Research also suggests that HDACs may be involved in transcriptional elongation by better allowing the association of enhancers and elongation factors, such as bromodomain-containing protein 4, to the appropriate region of DNA. More specifically, HDAC inhibitors enable RNA polymerase II to interact with negative elongation factor and attenuate the occupancy of bromodomain-containing protein 4 at promoters and enhancers [310]. These studies aid in our understanding of HDACs in promoting transcription and may guide future work attempting to unravel the molecular underpinnings of the placental HDAC9/*RGS2* relationship in preeclampsia.

### GPCR-associated kinases and oxidative stress as potential drivers of HDAC9 dysregulation in preeclampsia

Thus far, there is a general consensus that HDAC9 is repressed in preeclamptic placenta, but the underlying cause has yet to be discovered [34,40]. While the enzymatic and post-translational modifications of HDAC9 have been less extensively studied compared with others of its family, it is highly similar in sequence and structure. Thus, studies have been performed to explore analogous interactions and other inferences have been made based on conserved sites [88,90,91,95,96].

**Figure 5:**
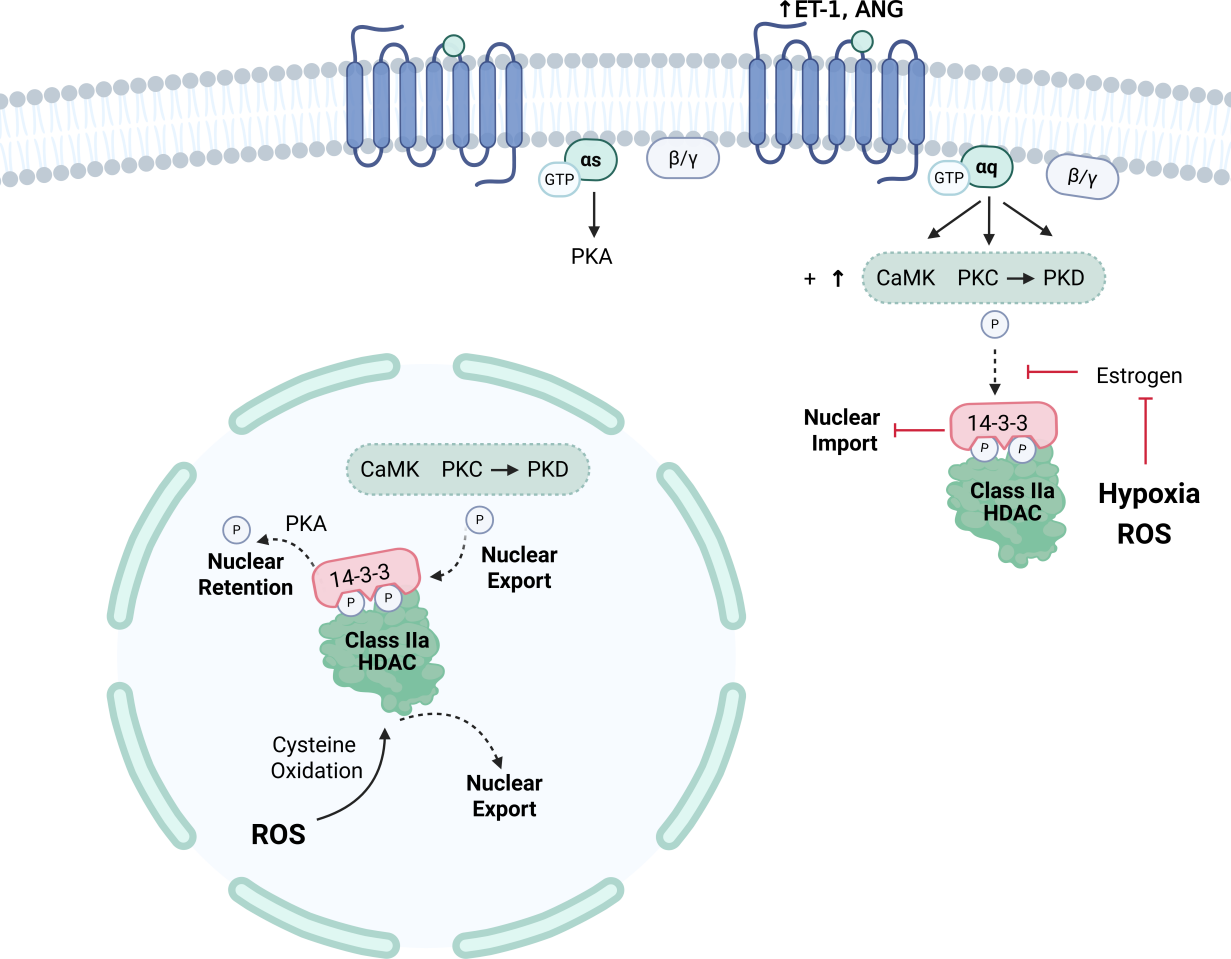
Proposed mechanisms of placental HDAC9 dysregulation in preeclampsia. The direct molecular mediators responsible for attenuated HDAC9 in the placenta during preeclampsia have yet to be explicitly determined. Nonetheless, HDAC9 has high sequence homology to others in its family and likely undergoes similar post-translational modifications to alter its cellular localization and activity [88]. The hormones ET-1 and ANG signal through the Gαq cascade, which leads to CaMK, PKC, and PKD activation [100,247,248]. These Gαq effectors promote HDAC phosphorylation, thus contributing to 14-3-3 docking and nuclear export or maintenance of a cytoplasmic localization where it can be degraded [82,86,88,89,94,95,100]. Hypoxia and excess ROS production may perpetuate lower placental estrogen [314–317], and estrogen is necessary to counteract the effects of ET-1 and ANG on HDACs [100]. Intranuclear ROS can also facilitate oxidation of cysteine residues on class IIa HDACs, contributing to their nuclear extrusion [311–313]. Contrarily, Gαs-mediated activation of PKA leads to HDAC dephosphorylation, prevents 14-3-3 binding, and promotes nuclear retention [82,88,97,98], but Gαq signaling may be more prominent in preeclampsia. ↑, upregulation; ↓, downregulation; ANG, angiotensin II; CaMK, Ca^2+^/calmodulin-dependent protein kinase; ET-1, endothelin-1; GTP, guanosine triphosphate; HDAC9, histone deacetylase 9; PKA, protein kinase A; PKC, protein kinase C; PKD, protein kinase D; ROS, reactive oxygen species.

Insight from class IIa HDACs suggests that kinases activated downstream of GPCR signaling [100], reduced estrogen [100], and intranuclear reactive oxygen species [311–313] have a complex interplay and contribute to HDAC9 dysregulation (Figure 5). As discussed above, many of the hormones implicated in preeclampsia, including angiotensin II [246,318,319], vasopressin [35,37], and endothelin-1 [32,320] signal through GPCRs [247,248], and their associated cascades can influence the distribution and activity of class IIa HDACs [91,94–96,100].

During propagation of the Gαq-mediated second messenger cascade, phospholipase Cβ (PLCβ) generates inositol 1,4,5-trisphosphate (IP₃) and diacylglycerol (DAG) [248]. DAG activates protein kinases, including conventional and novel PKC isoforms, and contributes to downstream activation of protein kinase D (PKD) [321]. Meanwhile, IP₃ induces calcium release from the endoplasmic reticulum, and the resulting Ca²^+^/calmodulin complex activates Ca²^+^/calmodulin-dependent kinases (CaMKs) [248]. Collectively, CaMKs [88,94], PKCs [91,95], and PKD [88,95] can phosphorylate class IIa HDACs, including the HDAC9 splice variant MITR, contributing to 14-3-3 docking. 14-3-3 binding was initially thought to evoke nuclear export, but it has since been recognized that it can also prevent nuclear re-import. Regardless, these events promote a cytosolic localization (where HDACs can be sequestered or degraded) [94,95,322,323] and impede their ability to repress transcription via changes in chromatin configuration [82,86,88,89]. Conversely, PKA, a Gαs effector, has opposing actions and supports nuclear retention of HDAC9 in cardiomyocytes by preventing phosphorylation at 14-3-3 binding sites [96]. There is indirect evidence of decreased PKA in preeclampsia, where reduced expression of CMIP in extravillous trophoblasts and syncytiotrophoblasts has been connected to up-regulation of PDE7B, lowering intracellular cAMP levels and potentially impairing PKA activation [324].

Research by Pedram et al. revealed that angiotensin II and endothelin-1 serve as modulators of HDAC4 and 5 [100], which are similar in structure to HDAC9 and belong to the same class [325]. More specifically, angiotensin II and endothelin-1 inhibited cardiomyocyte mRNA and protein synthesis of HDAC 4 and 5; they also promoted nuclear extrusion via phosphorylation, and these effects were countered by 17-β-estradiol [100]. Follow-up experiments indicated that angiotensin II-mediated phosphorylation of HDAC4 was calcium-dependent, and calcium signaling is exacerbated in preeclampsia—both evidenced by placental transcriptomic signatures [36] and enhanced calcium responses to angiotensin II in platelets [326]. On the other hand, Pedram et al. showed HDAC5 phosphorylation was facilitated by PKCδ and PKD [100]. PKCδ is elevated in preeclamptic placenta [327] and a recognized modulator of angiotensin II-induced PKD activation [328]. Regardless of the kinase involved, estrogen prevented both phosphorylation events [100].

While estrogen is fundamental for maintaining placental vascularization and trophoblast function [329], some studies report lower circulating and placental estrogen levels in preeclamptic patients [330–332]. The cause of decreased estrogen in preeclampsia has not been directly identified but may be a result of elevated reactive oxygen species [332] due to angiotensin II type 1 receptor autoantibodies (AT1AA) [74,318], endothelin-1 [333], vasopressin [35], or hypoxia [225]. A high oxygen environment within primary trophoblast culture induced aromatase activity, whereas low oxygen elicited the opposite effect [314]. Likewise, *aromatase* mRNA was reduced by utero-placental ischemia via ligation of the uterine spiral artery [315]. Addressing the oxidative stress component, hydrogen peroxide (H_2_O_2_) decreased estrogen receptor alpha (ERα) protein expression within breast cancer cells [316,317], but overexpression of an H_2_O_2_ scavenger prevented this downregulation [317]. If the same angiotensin II/endothelin-1/estrogen/HDAC9 relationship as revealed by Pedram et al. [100] exists in placental cells, reduced estrogen could lead to decreased nuclear localization of HDAC9, thereby attenuating its chromatin-modifying abilities.

Independent of GPCR-facilitated control of HDAC9, conserved cysteine residues within class IIa HDACs are subject to intranuclear oxidation, which provokes nuclear export [311–313]. Considering the wealth of evidence suggesting elevated oxidative stress and impaired antioxidant defenses in preeclamptic placental tissue [140,334–336] and animal models of the disorder [141,337], it is plausible that reactive oxygen byproducts contribute to HDAC9 dysregulation in women with this syndrome and may work in conjunction with GPCR effectors. More explicitly linking the two, intranuclear reactive oxygen species may arise as a byproduct of excess GPCR signaling, particularly Gαq activation. Angiotensin has been shown to stimulate NOX4-mediated oxidative stress in podocytes [338], and placental NOX4 protein expression is upregulated in preeclampsia [339]. Within cardiomyocytes, α1 adrenergic receptor activation (classically Gαq coupled) caused Nox4-dependent production of nuclear superoxide, which was responsible for nuclear extrusion of HDAC4. This redox-sensitive mechanism of HDAC4 translocation appears to be independent of CaMKII signaling and may instead be driven by NF-κB–mediated transcriptional regulation of NOX4 [313]. Supporting this, syncytiotrophoblast-specific Gαq stimulation in mice has been associated with elevated circulating levels of chemokines and cytokines known to be protein products of NF-κB target genes, suggesting a potential placental origin for these pro-inflammatory signals [36].

Combining these findings, if HDAC9 is necessary for *RGS2* transcription, a loss of HDAC9 would allow disinhibited GPCR signaling, particularly that of Gαq. This activation would permit elevated levels of reactive oxygen species, PKC, and calcium, which may further suppress HDAC9. However, this hypothesized feedback system has yet to be fully examined in placental cells. If this notion holds true, detecting and correcting aberrant hormonal, oxidative, or GPCR input poses a potential avenue to restore proper HDAC9 activity.

## Therapeutic potential and risks of HDAC9 activation in preeclampsia

While increasing HDAC9 expression or activity in trophoblast cells offers another therapeutic approach, achieving targeted delivery is crucial to minimize the maternal off-target effects of systemic activation. For instance, HDAC9 may increase the risk of vascular complications as HDAC9 has been implicated in aortic smooth muscle calcification [59], endothelial-mesenchymal transition [340], and increased aortic plaque lipid content in a mouse model of atherosclerosis [340]. Furthermore, though HDAC9-mediated promotion of migration, invasion, and angiogenesis is beneficial in the context of placental development, aberrant expression elsewhere may promote tumorigenesis (refer to Yang, et al. for a review of the role of HDAC9 in cancer) [54]. Leveraging similarities among the placenta and solid tumors, tumor-homing peptides have been used to facilitate targeted delivery of cargo to the placenta, with human placental explants showing accumulation within the syncytiotrophoblast. In mice, a combination of peptide-coated liposomes was used to facilitate administration to various placental regions with minimal uptake in off-target organs, aside from transient uptake in the liver and spleen that was reduced after 72 hours [341]. Thus, utilization of a similar strategy could be explored to enhance HDAC9 expression in syncytiotrophoblasts via pharmacological (i.e. small molecule activators) or biological (i.e. mRNA delivery, gene editing) approaches. HDAC9-specific activators are not yet available, but a small-molecular activator for HDAC8 exists (TM-2–51) [342] and could provide a foundation for developing targeted activators for other HDACs.

## Conclusions and future directions

In summary, preeclampsia is a multidimensional disorder consisting of placental, vascular, and immune dysfunction [15,62,343–345], and recent research suggests that HDAC9 is a significant molecular contributor [34,40]. Integrating newfound evidence from preeclampsia-focused studies with those of other disease models reveals a complex interplay between HDACs and relevant signaling pathways [51,61,100,167,311,313], which may control the activity of HDAC9 and its regulation of downstream targets. However, this review only begins to dissect some of the known and putative targets of HDAC9 relevant to preeclampsia, with a vast number likely still undiscovered.

Currently, our knowledge of HDAC9 in preeclampsia is restricted to ex vivo human placental tissues [34,40], in vitro cell culture experiments [34,40], and data extrapolated from related disease states [51,60,61,167]. Notably, no existing studies directly assess the precise role of HDAC9 in placental development across gestation or its involvement in the presentation of systemic features of preeclampsia, with maternal HDAC9 expression also remaining unexplored. Given i) preeclampsia is a heterogeneous disorder with multiple molecular subclasses [17] ii) despite this, the placenta is a key driver in the pathogenesis [26,346,347] iii) placental HDAC9 is down-regulated in preeclampsia [34,40] and iv) HDAC9 is an expansive modulator of related cellular processes including placentation, immunity, angiogenesis, and GPCR signaling [34,40,42,46,86,131,167,212,348], more comprehensive explorations are warranted. Extending these investigations to identify the precise role and targets of HDAC9 within specific placenta cell types as well as the upstream regulators responsible for disrupted HDAC9 in preeclampsia may provide unique perspectives in the field. Applying and advancing this knowledge has the potential to not only profoundly impact our understanding of this complex disorder but also to inspire new diagnostic and therapeutic avenues.

## Open Access

This article has been published open access under our Subscribe to Open programme, made possible through the support of our subscribing institutions, learn more here: https://portlandpress.com/pages/open_access_options_and_prices#conditional

## Abbreviations

GPCR: G protein coupled receptor
HDAC9: histone deacetylase 9
PlGF: Placental Growth Factor
RGS2: Regulator of G protein Signaling-2
sFLT1: Soluble FMS-like tyrosine kinase-1
STB: syncytiotrophoblast
tBHP: tert-butyl hydroperoxide
TIMP3: tissue inhibitor of metalloproteinases 3
VEGF: Vascular Endothelial Growth Factor
